# Treatment of Intracranial Vasospasm Following Subarachnoid Hemorrhage

**DOI:** 10.3389/fneur.2014.00072

**Published:** 2014-05-20

**Authors:** Andrew M. Bauer, Peter A. Rasmussen

**Affiliations:** ^1^Cerebrovascular Center, Cleveland Clinic Foundation, Cleveland, OH, USA

**Keywords:** cerebral vasospasm, balloon angioplasty, subarachnoid hemorrhage, cerebral aneurysm, delayed ischemic neurologic deficit

## Abstract

Vasospasm has been a long known source of delayed morbidity and mortality in aneurysmal subarachnoid hemorrhage patients. Delayed ischemic neurologic deficits associated with vasospasm may account for as high as 50% of the deaths in patients who survive the initial period after aneurysm rupture and its treatment. The diagnosis and treatment of vasospasm has still been met with some controversy. It is clear that subarachnoid hemorrhage is best cared for in tertiary care centers with modern resources and access to cerebral angiography. Ultimately, a high degree of suspicion for vasospasm must be kept during ICU care, and any signs or symptoms must be investigated and treated immediately to avoid permanent stroke and neurologic deficit. Treatment for vasospasm can occur through both ICU intervention and endovascular administration of intra-arterial vasodilators and balloon angioplasty. The best outcomes are often attained when these methods are used in conjunction. The following article reviews the literature on cerebral vasospasm and its treatment and provides the authors’ approach to treatment of these patients.

## Introduction

Cerebral aneurysmal rupture leading to subarachnoid hemorrhage is reported to occur at a rate of 5–8 per 100,000 annually, with a peak in incidence in the fifth decade of life (1). The first peak in morbidity and mortality comes with the aneurysmal rupture and ensuing brain damage and hydrocephalus. Treatment with urgent surgery if there is intraparenchymal clot, or external ventricular drain placement to treat hydrocephalus and elevated ICP has significantly lowered morbidity and mortality in this initial period. Early treatment of the ruptured aneurysm by either surgical or endovascular methods to avoid further morbidity and mortality from re-rupture is also indicated (2, 3).

After the initial subarachnoid hemorrhage, patients are still at risk of developing further morbidity and mortality or delayed ischemic neurologic deficit (DIND; also referred to here as clinically significant/symptomatic vasospasm). Symptomatic vasospasm develops in 20–40% of subarachnoid hemorrhage patients and is one of the least understood components in their care (4). Strokes from vasospasm account for nearly half of the early deaths in patients who survive the initial subarachnoid hemorrhage and aneurysm treatment (5). Angiographic vasospasm following aneurysmal subarachnoid hemorrhage was first described in 1950 in the work of Reid and Johnson (6) and published the following year (7). The recognition that development of vasospasm may play a large role in surgical outcomes was recognized early (8) and substantiated in 1976 with a large series suggesting that patients fare better if surgically treated within the first 48 h (9). The benefits of avoiding surgery during peak risk times for vasospasm were further characterized in the 1990s with the International Cooperative Study on the Timing of Aneurysm Surgery (10) showing that surgery during the time of peak vasospasm leads to the worst outcomes. The development of endovascular techniques has favorably impacted this concept as endovascular interventions can be coupled with treatment of vasospasm and do not seem to carry a worse prognosis when performed within the high-risk period.

While the ultimate underlying mechanisms that cause vasospasm are poorly understood, it has been established that the risk of DIND is closely related to the size of the subarachnoid clot. In 1980, Fisher published the landmark paper establishing a classification system for subarachnoid hemorrhage patients that was able to predict their risk of developing DIND (11). Although still widely used in clinical practice, the Fisher classification was based on computed tomography performed in its infancy, but the added risk related to increasing amounts of subarachnoid or intraventricular blood has been verified by other studies (12). Other factors such as young age, smoking, drug abuse, and pre-existing hypertension have been thought to be risk factors for vasospasm, but these have not helped much in prediction models for vasospasm (5). It is possible that these antecedent factors may play a role in the severity of spasm and the response to treatment.

Given that DIND is one of the leading causes of morbidity and mortality after aneurysmal subarachnoid hemorrhage, it is not surprising that many strategies have been proposed to effectively deal with it. Thus far, some of these have proven fruitful, while others have not. This has culminated in the publication of The Guidelines for the Management of Aneurysmal Subarachnoid Hemorrhage in 2009 (2). While aneurysm treatment has continued to improve, treatment of vasospasm continues to be the clinical event that often frustrates neurosurgeons and neurocritical care physicians and can easily ruin a technically brilliant “save” in this patient population. The remainder of this chapter will summarize some of the strategies that have been proposed to combat this challenging clinical entity.

## Diagnosis

To adequately diagnose vasospasm, one must first be careful to differentiate between clinical and radiographic spasm. The gold standard radiographic test for diagnosis is cerebral angiography, however, this is an invasive and expensive test and it is not practical for daily surveillance in all cases. Up to 70% of patients with aneurysmal SAH show constriction of the cerebral arteries on angiography after post-bleed day 3, but only about 50% of these patients have a neurologic deficit attributable to this arterial distribution, and 20% of them will go on to develop infarction (2). This brings some controversy into the algorithm that should be used for vasospasm treatment or prophylaxis as many of the treatments carry some degree of morbidity themselves. In our practice, subarachnoid hemorrhage patients between day 3 and 14 who develop a new neurologic deficit not explained by rebleeding or hydrocephalus are taken for emergent angiography. High-grade patients with limited neurologic exam are more difficult and the index of suspicion must be kept higher.

Transcranial Doppler technology was developed in the 1980s for indirect measurement of vessel caliber by way of blood flow velocity. Given that it is an indirect measure of vessel diameter, its use is somewhat controversial and benefits are not entirely clear. In addition, the utility can be limited by patients with extremely thick temporal bone that limit ultrasound windows, systemic therapies such as HHH (discussed below), which alter hemodynamics, and inter-observer and institutional variability, which make it difficult to standardize across the general population. TCDs have been shown to be reliable in the MCA with sensitivity of 67%, specificity of 99%, positive predictive value of 97%, and negative predictive value of 78% (13). These values fall off significantly when looking at other brain vessels, again making TCDs less reliable in areas such as the posterior circulation. In 2004, the American Academy of Neurology conducted a systematic review of the literature and concluded that TCDs can be used reliably to screen for the presence of vasospasm in the MCA, but not other vessels (14). They also suggested the following criteria for the diagnosis or exclusion of vasospasm: flow velocity >200 or <120 cm/s, respectively, significant increase in the flow velocities from day to day (>50 cm/s), and Lindegaard ratio (MCA velocity/ICA velocity) >6. In our practice, we routinely obtain daily or every-other-day transcranial Doppler studies in these patients from the day of the bleed to post-bleed day 14 and use the values mainly to follow trends and as a warning sign.

CT angiography has also been used in some centers for the detection of cerebral vasospasm. Several small prospective cohorts have shown good correlation between CTA and DSA in predicting vasospasm and that many unnecessary angiograms could be avoided by using CTA as a screening test (15–17). A more recent meta-analysis found a sensitivity and specificity for CTA of 80 and 93%, respectively (18). It has been thought that adding CT perfusion, or another dynamic imaging modality to CTA would significantly increase its use as a screening study for cerebral vasospasm, but this has not necessarily been the case. One of the difficulties has been in which parameter of CTP to follow. Overall, meta-analysis has found a sensitivity of 74% and specificity of 93% of CTP in the detection of cerebral vasospasm (18). In our practice, we do not use CTA or CTP routinely in diagnosis of vasospasm but reserve its use for sporadic complex cases. We find it more efficient to move the patients with new deficits or large TCD changes directly to angiography as this is the most accurate diagnostic tool and also gives the option of treatment. This also serves to limit the amount of radiation and contrast the patient is exposed to.

## Prevention of DIND

The first step in the reduction of morbidity and mortality from vasospasm is prevention of DIND. Several preventive strategies have been proposed and studied and all are used with variable degrees of frequency in the care of subarachnoid hemorrhage patients today. Several excellent reviews are available detailing the trials that have been conducted (2, 19).

Treatment with oral Nimodipine, a calcium channel blocker, has become essentially standard of care in the United States for all patients with subarachnoid hemorrhage. This is based on the 1983 trial by Allen et al. (20) in which 13% of patients in the placebo group suffered a severe neurologic deficit related to vasospasm vs. 1.7% in the Nimodipine group (*p* < 0.03). A larger randomized trial was conducted in 1989 and showed reductions of 34% in ischemic stroke and 40% in poor outcome in patients treated with Nimodipine compared to placebo (21). It is thought, however that these results may be in some way related to cerebral protection since there has been no demonstration of reduction of angiographic spasm in patients on Nimodipine (2, 22). Ongoing trials using different preparations of Nimodipine are ongoing. Other agents such as nicardipine have not shown the same benefits when given intravenously (23). One promising new use for calcium channel blockers is through intrathecal administration. No large-scale studies have been conducted but the intrathecal administration of nicardipine has been shown in smaller studies to reduce TCD velocities within 8 h of administration (24). Nicardipine pellets have also been developed that can prevent local vasospasm after aneurysm clipping (25, 26). Clinical benefits of these therapies have not yet been firmly established.

There has been much interest in the potential of statins to reduce the morbidity and mortality of vasospasm. Statins are thought to improve cerebral vascular reactivity through cholesterol-dependent mechanisms. Much of this literature has stemmed from cardiology. Several clinical trials have been conducted using statins in SAH. Tseng et al. (27) found that SAH patients randomized to pravastatin had a 32% reduction in TCD-diagnosed vasospasm, as well as 83% reduction in vasospasm-related DIND and 75% reduction in mortality. A meta-analysis including this trial and the other RCTs of statin use in 158 SAH patients showed statistically significant reduction in vasospasm (RR = 0.73), DIND (RR = 0.38), and mortality (RR = 0.22) (28). More recent trials have failed to show such a robust benefit (29, 30).

The idea that DIND is caused by clotting in the spastic small cerebral vessels has led to some investigation of the merits of fibrinolytic agents in the treatment of SAH patients. A meta-analysis including five prospective trials and three retrospective series with historical controls found significant absolute risk reduction of 14.4% for DIND, 9.5% for poor GOS score, and 4.5% for death (31). The benefits to functional outcome, morbidity, and mortality have been offset by the high number of complications with this therapy and it has not gained widespread acceptance in clinical practice (32). This same line of thinking has led to trials with aspirin (33), enoxaparin (34, 35), and tirilizad (36, 37), but these therapies have not been shown to be effective in reducing vasospasm-related morbidity and mortality in subarachnoid hemorrhage.

Magnesium has also been studied as an agent to inhibit voltage-gated calcium channel contraction of vascular smooth muscle. The magnesium in aneurysmal subarachnoid hemorrhage (MASH) trial (38) randomized 283 patients to continuous IV magnesium infusion vs. placebo. This showed a trend toward lower delayed cerebral infarction (RR = 0.66) and poor clinical outcome at 3 months (RR = 0.77), but these findings failed to reach statistical significance. This finding was confirmed in a second trial, MASH-2, which also included a meta-analysis of 2047 patients, which also showed that magnesium was not superior to placebo for reduction of poor outcome after subarachnoid hemorrhage (39). It is our general practice in the ICU, however, to monitor magnesium and supplement to normal levels.

Perhaps the most promising of the new medical therapies for vasospasm are the endothelin receptor antagonists. Endothelin I, when bound to its receptor, is a potent activator of vascular smooth muscle cells resulting in vasoconstriction. Several trials have been conducted using Clazosentan (AXV-034343) (40) and TAK-044 (41) and all have shown significant decreases in angiographic vasospasm. Perhaps the most interesting study of clazosentan randomized 32 patients to continuous IV infusion of clazosentan vs. placebo and monitored for symptomatic, angiographically proven vasospasm (42). Patients in the placebo arm who developed vasospasm were allowed to cross over into the study group. There was 48% relative risk reduction in symptomatic vasospasm in the groups as designed, and 50% of the patients who crossed over had resolution of their vasospasm when treated with the study drug. In the intention-to-treat analysis, there was a trend toward decreased delayed cerebral infarction with clazosentan (15 vs. 44%, *p* = 0.13), but it failed to achieve statistical significance. It is promising; however, that the endothelin receptor antagonists may be a target not only for prevention of vasospasm, but also potentially a treatment once it has already developed. While there has been a clear trend toward decreased angiographic vasospasm in these studies, they have failed to show a clear benefit in outcomes and there has been a relatively high rate of pulmonary complications, hypotension, and anemia (19).

Ultimately, the only medical strategies for prevention of vasospasm with enough evidence to be included in the guidelines for SAH patients were maintenance of normal circulating blood volume (discussed below), and oral Nimodipine. Hopefully this will change in the future as further randomized trials are conducted using newer preventive therapies.

## Treatment of Vasospasm

The ultimate goal in the treatment of cerebral vasospasm after subarachnoid hemorrhage is to avoid DIND by reducing ICP, optimizing the rate of cerebral oxygen demand, and improving cerebral blood flow. Given these goals, early aneurysm treatment and ventriculostomy placement for patients with elevated intracranial pressure is a necessity. Early aneurysm treatment allows the treatment team to be more aggressive with further vasospasm treatment over the course of care.

HHH-therapy (hypertension, hypervolemia, and hemodilution) has been a mainstay in the treatment of SAH patients for many years. The idea that avoiding hypovolemia and hemoconcentration as a mechanism to improve outcome is not novel, and seems intuitive; nonetheless, taking an active roll in driving these parameters has been a little more controversial. This is especially true since the detrimental effects of this therapy include pulmonary edema, respiratory failure, cardiac failure, renal dysfunction, and exacerbation of cerebral edema. It is useful to break this therapy into its components for further analysis.

One of the heralding signs of vasospasm is often elevated blood pressure, with whatever is left of cerebral autoregulation attempting to increase cerebral blood flow by increasing systemic pressure. Whether induced hypertension is useful in preventing arterial vasospasm is another question altogether. One study showed that induced hypertension was able to achieve higher flow in ischemic, but not infarcted territories, despite no change in global CBF (43). No study of induced hypertension in isolation, though, has shown a decrease in the development of angiographic vasospasm. Thus, it is likely that hypertension may be useful in reversing neurologic deficits that develop from vasospasm, but not as a preventive mechanism by itself (44). In our practice, we allow the patient to auto-regulate the blood pressure to levels up to 180 or sometimes 200 systolic once the aneurysm is secured. If the patient develops symptomatic vasospasm with a systolic blood pressure lower than 180, then we usually augment the blood pressure with vasoactive medications.

Hypervolemia is perhaps the most controversial of the HHH components. Many centers continue to use hypervolemia, often dictated by the use of central venous and pulmonary artery catheters, despite the lack of evidence that it is beneficial. One study (45) randomized 82 patients to either hypervolemic or euvolemic status, which was maintained to day 14 after aneurysmal rupture. The central venous pressures were higher in the hypervolemic group, but here was no difference in cerebral blood flow or cerebral blood volume and the incidence of vasospasm was 20% in both groups. Another study found no difference in the incidence of vasospasm or in clinical outcome, but the hospital costs and complication rate were much higher in the patients treated with hypervolemia (46). There is little doubt to the fact that hypovolemia is detrimental to these patients, but hypervolemia may be detrimental as well (2). In our practice, we prefer euvolemia and make every effort to monitor the fluid status and patient weight closely.

Relatively little attention is given to hemodilution as a component of HHH-therapy. Many SAH patients become hemodiluted as a result of operative blood loss and aggressive fluid resuscitation. While hemodilution can increase local CBF by decreasing blood viscosity, it does so at the expense of severe decreases in oxygen delivery capacity (47). Other studies have shown that blood transfusion is an independent risk factor for poor outcome (48), however this finding may be the result of whatever insult caused the need for blood transfusion. The guideline authors did not find sufficient evidence to make a recommendation either way (2). In our practice, we generally keep the hematocrit around 30%, and use clinical indicators such as tachycardia or signs of decreased oxygen delivery as indications for transfusion.

## Endovascular Management of Vasospasm

Endovascular balloon angioplasty techniques for treatment of cerebral vasospasm were first described in 1984 (49). The further development of these techniques in the treatment of vasospasm is attractive as it may improve outcomes and ameliorate the detrimental effects of aforementioned HHH-therapy. There is still much controversy today, however, regarding which techniques are best, which patients should be candidates, and the best time to intervene. Overall, the quality of evidence for intra-arterial therapies is low, but it has gained nearly universal acceptance in the algorithm for SAH treatment in centers that have quality endovascular services.

Some controversy exists as to the specific timing of endovascular therapy. One randomized trial (50) has been conducted on the use of balloon angioplasty as a prophylactic measure. Patients treated with prophylactic balloon angioplasty had a significant decrease in the need for urgent rescue therapy for symptomatic vasospasm (12 vs. 26%, *p* = 0.03) but had no statistical difference in the rate of cerebral infarction (23.5 vs. 31.8%, *p* > 0.05) or poor outcome at 3 months (relative risk reduction 29.4%, *p* > 0.05). Several studies have shown that patients had better neurologic improvement if the intervention (angioplasty or intra-arterial vasodilator administration) is performed as urgently as possible after the neurologic decline with vasospasm (51, 52). The risk of complications must be taken into account when determining the timing of intervention. In the prophylactic intervention study, 4/85 (4.7%) patients had vessel perforations leading to death in three (50).

The choice of therapy is largely dictated by the presumed pathology. If there is little angiographic spasm in the carotid siphon or M1 segment, then intra-arterial administration of vasodilators is the best therapy. Studies have been conducted using many different drugs, but many are small and retrospective. In 1993, Kassell et al. (53) described the intra-arterial administration of papaverine for the treatment of vasospasm and showed marked angiographic improvement in 66% and clinical improvement in 33%. These findings have been replicated in other studies (54). Papaverine was reported to be neurotoxic and resulted in neurologic decline in one study (55) and is very rarely used today in favor of calcium channel blockers. Verapamil (56, 57) and nicardipine (58, 59) have also been used successfully by intra-arterial administration in the treatment of vasospasm. The exact protocol for the dosing and delivery of these agents is not clear. Some prefer a long, slow administration time, while others give the medication as a bolus. Interestingly, it has also been described to administer intra-arterial verapamil via an indwelling microcatheter in the treatment of refractory vasospasm (60). This method could easily be complicated by thromboembolic events, however. All of these agents are vasodilators and administration should result in increased CBF and CBV, therefore, it is recommended that ICP be monitored during treatment (2). It is also important to monitor for systemic hypotension, as this may be more detrimental than the vasospasm itself. In our practice, we use verapamil and nicardipine in 10 mg aliquots in each vessel. At times, if there is no systemic hypotension it is reasonable to increase to 20 or 30 mg in divided doses if the spasm is severe. We have also seen better and somewhat more durable results when the 10 mg dose is infused slowly on a pump over 10–20 min, although this method may not be possible depending on the stability of the patient.

**Figure 1:**
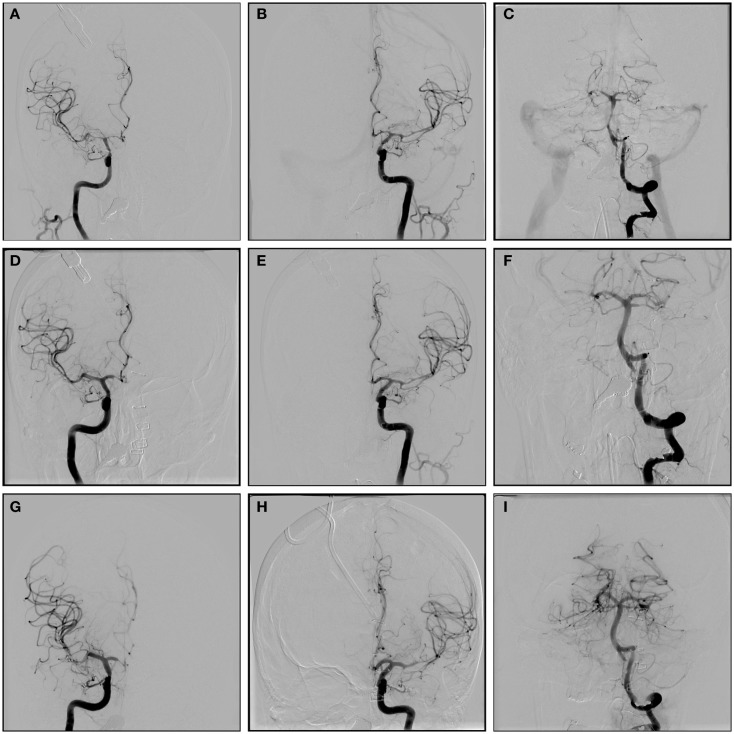
**A-P angiographic images of a 58-year old woman with Hunt and Hess grade V subarachnoid hemorrhage from ruptured dissecting right vertebral aneurysm**. Concern for vasospasm was generated from routine transcranial Doppler testing. The patient was already maximally medically managed so was taken for angiography. A-P images of RICA **(A)**, LICA **(B)**, and basilar artery **(C)** showing very severe vasospasm. A-P images of RICA **(D)**, LICA **(E)**, and basilar artery **(F)** immediately after balloon angioplasty and administration of intra-arterial verapamil showing resolution of the spasm in the ICA, M1, A1, and basilar artery, as well as improvement in the more distal spasm. A-P images of the RICA **(G)**, LICA **(H)**, and basilar artery **(I)**, 6 days after original treatment showing durability of the angioplasty treatment in the ICA, A1, M1, and basilar artery, but recurrence of spasm in the more distal vessels. This was treated with administration of verapamil.

In the larger cerebral vessels (ICA, M1, and basilar), balloon angioplasty has been shown to be very effective and may be more durable. Angioplasty is not generally considered to be safe beyond the carotid or M1 segments (50), although this thought may change with the introduction of newer balloon catheters that are safer in more distal segments. We use angioplasty sparingly in the A1 segment. Overall, the current literature regarding balloon angioplasty for vasospasm is somewhat sparse in terms of quality data. One study compared the effectiveness of balloon angioplasty to intra-arterial Nimodipine and found both therapies to be effective in radiographic resolution of vasospasm, but no difference in clinical outcomes (61). The only high-quality data regarding angioplasty for vasospasm comes from the prophylactic balloon angioplasty trial mentioned above (50). This trial showed a significant decrease in DIND as well as need for therapeutic angioplasty, suggesting the durability of this treatment. A few other smaller trials have shown similar trends, but are significantly limited by small sample size (62). Further clinical trials in this area are necessary to fully investigate the benefits of balloon angioplasty for cerebral vasospasm. In our institution, we favor balloon angioplasty when moderate to severe vasospasm is seen in the large vessels (ICA or M1), but we always use this in conjunction with intra-arterial vasodilator therapy (Figure 1), to adequately treat spasm in the more distal vessels.

## Conclusion

Cerebral vasospasm is an important source of morbidity and mortality in subarachnoid hemorrhage patients. Aggressive ICU care and compulsive management style are necessary to adequately manage these patients. The body of literature on cerebral vasospasm is relatively well developed, but still subject to the relative heterogeneity and complexity of this group of patients. The only Class I evidence regarding cerebral vasospasm used in the publication of the AHA subarachnoid hemorrhage guidelines (2) was that in favor of oral Nimodipine. Early aneurysm treatment, HHH-therapy, cerebral angioplasty, and selective intra-arterial vasodilator therapy was recommended based on Class II evidence. Many other pharmacologic and interventional strategies are currently being investigated. There is little doubt that reduction of DIND will go a long way in reducing the overall morbidity and mortality of subarachnoid hemorrhage patients, and significantly improve our ability to care for them in the future.

## Conflict of Interest Statement

The authors declare that the research was conducted in the absence of any commercial or financial relationships that could be construed as a potential conflict of interest.
